# Can Topical Glyceryl Trinitrate be Effective in the Treatment of Levator Ani Syndrome?

**DOI:** 10.5152/tjg.2025.24489

**Published:** 2025-01-13

**Authors:** Fırat Canlıkarakaya, Serhat Ocaklı

**Affiliations:** 1 Department of General Surgery, Amasya University Faculty of Medicine, Amasya, Türkiye; 2 Department of General Surgery, Ankara Medipol University Faculty of Medicine, Ankara, Türkiye

**Keywords:** Glyceryl trinitrate, levator ani syndrome, proctalgia, Rome IV

## Abstract

**Background/Aims:**

Levator ani syndrome (LAS) is a pathology that is both difficult to diagnose and treat. The effectiveness of current treatments, which are mostly administered with special devices in advanced healthcare centers, is controversial. The aim of the study is to investigate the effects of glyceryl trinitrate, which is easily accessible and can be applied by the patient, in the treatment of LAS.

**Materials and Methods:**

The study cohort comprised 16 patients with LAS diagnosed and received 0.4% topical glyceryl trinitrate treatment rectally.

**Results:**

At the first month of treatment, it was observed that the symptoms and puborectal muscle tension of 15 (93.7%) patients had completely resolved. One patient with persisted symptoms was referred to an advanced center. Symptomatic relief continued in 14 (87.5%) of the 15 patients at the third month of treatment and there were no pathological findings in the rectal examination. The visual analog scale score significantly decreased after the treatment (*P *< .001).

**Conclusion:**

It is thought that topical glyceryl trinitrate treatment may be at least as effective as current treatment methods. Although the number of patients is small, our study is inspiring as it is the first time this molecule has been tried in the treatment of LAS in the literature. More comprehensive randomized controlled studies with long-term follow-up of patients are needed.

**Main Points**

- Topical application of glyceryl trinitrate may be effective in the treatment of levator ani syndrome.
- The success rate after 3 months of treatment is 87.5%, and the recurrence rate at 6 months after completion of treatment is 9%.
- Topical glyceryl trinitrate treatment can be applied by the patient at home without the need for a special device, it is cheap and easily accessible.

## Introduction

Proctalgia syndrome is a clinical condition that causes pain in the anal region, and no organic pathology is observed to cause the pain. Levator ani syndrome (LAS) is a pathology that is evaluated among proctalgia syndromes and is more clearly defined by the latest 2016 Rome IV criteria.^1^ The structure called levator ani anatomically is the name given to all of the puborectalis, pubococcygeus, and iliococcygeus muscles. This structure supports the pelvic floor and takes part in defecation, urination, and sexual functions.^2^ The pathophysiology of LAS has not been fully revealed. The most widely accepted view is that the puborectal muscle cannot relax properly after spastic contractions, resulting in compression of the blood vessels and pain due to ischemia.^3^ The typical clinical manifestations of this syndrome are the presence of rectal pain and pressure lasting more than 30 minutes, the spread of this condition to the thigh and vaginal area, the triggering of this condition by defecation, stress, and sitting for a long time, and the pain disappearing on its own.^1^

It is very difficult to diagnose this pathology as every functional disease. There is no standard examination for diagnosis. While the absence of any organic pathology that would cause this clinical condition is the most important criterion in diagnosis, the presence of tenderness and tension in the puborectalis muscle on rectal examination is also a supportive finding for this syndrome.^4^ A good anamnesis taken from the patient is the best guide. The diagnosis can be supported by rectal inspection and touch.^2^ In order to standardize patients and make the diagnosis more clear, the diagnostic criteria of the Rome IV guideline are frequently used today. Accordingly, chronic or recurrent rectal pain lasting longer than 30 minutes and tenderness during traction on the puborectalis muscle must be present, and other causes of rectal pain such as inflammatory bowel disease, intramuscular abscess, anal fissure, thrombosed hemorrhoids, prostatitis, coccygodynia, and major structural alterations of the pelvic floor must be excluded. All of these criteria must be met, and the symptoms must have started at least 6 months before diagnosis, and the criteria must have been met for the last 3 months.^5^ Although there is no clear information due to the fact that it is a functional disease and the difficulties in diagnosis, the incidence of LAS has been reported in the literature at rates ranging between 1.7% and 6%.^6,7^

Treatment is as challenging as diagnosis in functional intestinal and proctological pathologies. This is why there is a wide range of treatments. There are also many treatment options for LAS. Sitz baths and pelvic floor muscle exercises are conservative treatment methods that have been used for years.^8,9^ Botulinum toxin injection and electrogalvanic stimulation are other current treatment methods.^10^ Biofeedback therapy is another method that has a place in treatment.^11^ Sacral nerve stimulation and some surgical methods are also used in LAS treatment.^1^

Glyceryl trinitrate, also known as trinitroglycerin, is a vasodilator agent. Nitric oxide, released after metabolism, acts as a vasodilator in low doses and an arterial dilator in high doses.^12^ After its first discovery in 1847, its journey began with the treatment of angina pectoris and continued with its use in many cardiovascular pathologies such as acute hypertension and pulmonary hypertension.^13-15^ Its main metabolite, nitric oxide, also has a relaxant effect on striated muscle tissue. In this respect, glyceryl trinitrate, also used in the treatment of anal fissure, reduces the need for surgical intervention.^16-18^ Additionally, it is also among the treatment options for striated muscle pathologies such as Achilles tendinitis and patellar tendinitis.^19^

A retrospective study was planned to investigate the effect of glyceryl trinitrate, already known to have antispasmodic effects and is used safely in the perianal region, on LAS, which is thought to develop as a result of puborectal muscle spasm.

## Materials and Methods

The study was approved by the Ethics Committee of Ankara Etlik City Hospital (approval number: AEŞH-BADEK-2024-136; date: April 24, 2024). Since the study was retrospective, an informed consent form was not prepared.

### Patient Selection and Study Design

The data of 16 patients who were diagnosed with LAS and treated rectally with 0.4% topical glyceryl trinitrate between August 1, 2022, and July 1, 2023, were retrospectively examined. Clinical information of all patients was determined from the hospital registry system.

Patients over the age of 18 were included in the study. Rome IV criteria were used for the diagnosis of LAS. It was taken into consideration that there was no organic pathology that could explain this situation in the total colonoscopy of patients over the age of 40 within the last 1 year. Rectal examinations of all patients were performed, and it was taken into consideration that there was no organic pathology that could explain this situation. About 0.4% of topical glyceryl trinitrate was applied rectally to all patients, every 12 hours, twice a day. Patients were not given a special diet other than a high-fiber diet and plenty of water consumption. It was confirmed that the patients were not using drugs that affect the digestive system, such as antidepressants or laxatives. The patients were re-evaluated at first and third months of treatment. Then, the treatment was terminated, and the patients were re-evaluated after 6 months. During the follow-up examinations, the regression, decrease, or disappearance of the patients’ complaints were questioned. The change in puborectal muscle sensitivity and tension was evaluated during rectal examination. Visual analog scale (VAS) evaluation was also performed before and after treatment.

### Statistical Analysis

Categorical variables such as age, gender, and treatment success were expressed as the number of patients (frequency) and percentage (%). The change in VAS score before and after treatment was evaluated by the Wilcoxon signed-rank test.

All analyzes were performed with the SPSS version 25 (IBM SPSS Corp.; Armonk, NY, USA) package program.

## Results

It was observed that the symptoms of 15 of the 16 patients included in the study disappeared completely and the tension in the puborectal muscle normalized during rectal examination at the first month of follow-up. One patient whose symptoms did not subside and levator ani muscle tension persisted during examination was referred to an advanced center. Symptomatic relief continued in 14 of the 15 patients who were followed up at the third month of treatment, and there were no pathological findings on examination. One patient stated that his symptoms had started again; no pathological findings were detected in the rectal examination, and he was referred to an advanced center for alternative treatment methods. Six months after completion of treatment, 3 patients did not attend follow-up appointments regularly. During the 6-month medication-free follow-up, 10 of the 11 patients remained asymptomatic, whereas 1 patient reported a recurrence of pain upon discontinuation of the medication.

As a result of the study, the success rate of the treatment at the first and third months were found to be 93.7% and 87.5%, respectively. Among the 11 patients who responded positively to a 3-month treatment regimen and were followed for an additional 6 months, 91% experienced no recurrence.

The median value of the VAS scores before treatment, third month of treatment, and 6 months after treatment were 6 (min: 3, max: 8), 1 (min: 0, max: 8), and 0 (min: 0, max: 0), respectively. This change in VAS score was found to be statistically significant (*P *< .001) (Table 1).

## Discussion

This study is a pioneering investigation that demonstrates the potential efficacy of glyceryl trinitrate in the treatment of LAS.

Although LAS is seen at a low rate of 1.7%, it leads to recurrent outpatient clinic visits and a corresponding increase in workforce loss and healthcare expenses due to difficulties in making a clear diagnosis. Difficulties in diagnosis and treatment lead to patient accumulation in advanced centers.

There are conservative methods available for the treatment of LAS that have been tried for a long time. Since the disease is functional by prioritizing its psychogenic basis, it is recommended that the patient soothe bad emotions. Apart from this, warm sitz baths and non-rigid sit rings have also been used, but the effectiveness of these treatment approaches is quite limited.^20 21^ There are also studies on the use of tricyclic antidepressants and diazepam in the literature, but there is no clear information about success rates. Considering the addiction and other side effects, the place of these treatments in routine practice is limited.^22,23^

Non-surgical methods are the primary choice in the treatment of LAS. In a study including 76 patients comparing transperineal botulinum toxin application with electrogalvanic stimulation, 44.7% of the patients who received botulinum toxin injection and 57.1% of the patients who received electrogalvanic stimulation did not benefit from the treatment.^10,24^

Biofeedback application is a frequently used treatment method in functional anorectal diseases. It is based on the principle of training the patient’s own muscles with assistive technological products. Biofeedback application is preferred as the first-line treatment method in most centers because it provides symptomatic relief of up to 87% in studies examining its success in LAS treatment.^25^

Although there is no data on the use of sacral nerve stimulation only in the treatment of patients diagnosed with LAS, there are studies conducted on patients with chronic idiopathic anal pain including these patients. In a study conducted with 12 patients, it was observed that there was a decrease in the pain scores of the patients, although there was no complete recovery.^26^ The translumbosacral neuromodulation method, which is a more specific form of stimulation, was tested in patients diagnosed with LAS, and 32 LAS patients and 31 control patients were examined. In this study, symptomatic improvement was observed in 13 patients.^27^

There are not many studies in the literature on surgery in the treatment of LAS. In a study, lateral internal sphincterotomy was performed on patients as a surgical method, and no benefit was obtained in any of the patients.^28^ In the literature, partial puborectal muscle resection was performed on a population including LAS patients, and none of the patients were cured. Moreover, serious complications such as gas and fecal incontinence developed in these patients.^29^

In light of the above information, it can be said that surgical and medical treatments for the treatment of LAS are far from success due to low efficacy, serious complications, and side effects. Methods such as botulinum toxin injection, electrogalvanic stimulation, and sacral nerve stimulation have low success rates and are not accessible as they require repeated applications with the help of special devices in advanced centers. Applications such as sacral nerve stimulation and biofeedback therapy, which seem to be more successful, also require repeated applications with special devices in advanced centers. These methods have low accessibility and high costs.

Topical glyceryl trinitrate, used in this study, is a relatively cheap, easily accessible medicine that can be easily applied by patients at home. Moreover, given the high efficacy rates of 93.7% and 87.5% at the first and third months of treatment, respectively, and the absence of recurrence in 91% of patients followed up for 6 months post-treatment, it is suggested that this treatment modality may have a role in the management of LAS. Despite the small sample size, this study is inspiring due to its pioneering investigation of this molecule in LAS treatment, coupled with high treatment success rates and a low recurrence rate.

Topical glyceryl trinitrate, which was found to provide symptomatic relief at a higher rate than other treatment methods in the literature, was considered to be a medicine that could be prioritized among treatment options. Other important advantages of this treatment are that it can be applied by the patient at home without the need for a special device, and it is cheap and easily accessible. More comprehensive randomized controlled studies with long-term follow-up of patients are needed.

## Figures and Tables

**Table 1.** Change in VAS Score with Treatment

		Median	Standard Deviation	*P*
VAS score	Before treatment	6	1.42	<.001*
	Third month of treatment	1	1.96	
	Six months after treatment	0	0	

VAS, visual analog scale.

feedbacknerve stimulation*Wilcoxon signed-ranks test.

## Data Availability

The data that support the findings of this study are available on request from the corresponding author.
